# Measuring and optimizing spatial accessibility to emergency medical services with a focus on age-based spatial variations in EMS risk

**DOI:** 10.3389/fpubh.2026.1827404

**Published:** 2026-06-09

**Authors:** Weicong Luo, Xinxin Chen, Luyan Zhao, Lijie He

**Affiliations:** College of Public Administration, Huazhong Agricultural University, Wuhan, China

**Keywords:** accessibility, age groups, EMS risk, GIS, spatial optimization

## Abstract

**Introduction:**

Emergency Medical Services (EMS) play a crucial role in safeguarding public health, yet their efficiency is highly dependent on spatial accessibility and optimal facility location. Existing research often evaluates EMS spatial accessibility using total population counts, overlooking the heterogeneity of EMS risk across space and age groups. This study aims to improve EMS accessibility measurement and spatial optimization by integrating spatial and demographic variations in EMS risk.

**Method:**

An integrated framework combining a modified two-step floating catchment area (2SFCA) model and two spatial optimization models—the Maximum Covering Location Problem (MCLP) and the P-median model—were developed. The improved models incorporate spatial and age-based EMS risk derived from 2024 EMS records in Wuhan, China. Comparative analyses were conducted between traditional and risk-integrated models to examine differences in accessibility and optimal station layouts.

**Results:**

Findings reveal significant age-related and spatial variations in EMS risk, with older adults populations showing markedly higher risks, and high-risk areas concentrated in central and near-suburban districts. Compared to traditional 2SFCA results, the improved model produced significantly different accessibility distributions, particularly in peri-urban areas. In optimization analyses, risk-integrated MCLP and P-median models located new EMS stations closer to near-urban areas, whereas traditional models favored peripheral suburbs.

**Conclusion:**

Incorporating spatial and demographic variations in EMS risk substantially enhances the accuracy of accessibility measurement and optimization outcomes. The results underscore the importance of prioritizing high-risk older adults populations, strengthening EMS capacity in urban cores, and improving coverage in underserved suburbs. This framework offers a transferable tool for developing more efficient and equitable EMS systems in rapidly urbanizing and aging regions.

## 1 Introduction

Emergency Medical Service (EMS) is a vital component of the healthcare system, providing urgent medical care and transportation for patients experiencing sudden illness or injury. The nature of EMS is highly time-sensitive, and even a slight delay can directly and substantially affect patients’ survival rates (1, 2). To ensure high-quality services and efficient operation of EMS, health planners typically assess spatial accessibility and optimize the spatial layout of medical resources to improve system performance (3–5), all of which are predicated on a thorough understanding of local EMS demand characteristics. Generally, EMS potential demand is often determined by population size and EMS risk (that is, the annual probability that an individual will require EMS). Precisely characterizing the spatial distribution of EMS demand constitutes a fundamental prerequisite for conducting accessibility assessments and spatial optimization analyses.

To identify the nature of distribution of demand, it is necessary to understand the EMS risk, which shows substantial variation across both spatial and demographic dimensions. On the one hand, EMS risk exhibits significant spatial variation, primarily influenced by factors such as environmental pollution, built environment, and socioeconomic conditions within different areas (6–9). For example, McLeod et al. (8) found that the area with poor air quality is significantly associated with an increase in EMS call volumes. On the other hand, EMS demand risk is closely related to the age structure of patients. Different age groups display obvious differences in health status, disease profiles, physical function, and the ability to cope with acute health events, leading to varying degrees of reliance on and demand for EMS. Many studies found that with the same level of medical resources, areas with a larger older adults population tend to have higher EMS call rates due to age-related declines in physical function and a higher prevalence of chronic diseases (10–12). Therefore, prior to studying EMS accessibility and spatial optimization, it is important to consider variations in EMS risk across both spatial patterns and age structures, so as to better capture the demand characteristics of the study area.

Due to the time-sensitive nature of EMS, this study focuses primarily on spatial potential accessibility to EMS, defined as the ease with which an EMS team can reach the scene of an incident. Differences in healthcare accessibility primarily result from spatial and non-spatial factors (13). Over the last decades, various spatial methods have been developed to measure EMS accessibility, such as proximity-based or gravity-based methods (14). Recently, accessibility measurements have also been refined along multiple dimensions (e.g., traffic modes, real-time congestion, and distance decay functions) to yield more accurate results (3, 15, 16). To enhance accessibility, spatial optimization models are widely employed, which integrate mathematical formulas with Geographic Information Systems (GIS) to identify the optimal spatial arrangement of facilities or resources across geographic space under specified objectives and constraints (17). In recent years, spatial optimization models have been widely adopted in EMS planning, serving as powerful tools to address issues such as facility location, resource allocation, and service coverage (4, 18–20).

However, the limitation of existing studies related to EMS accessibility and spatial optimization is that most scholars still evaluate EMS demand with total population counts or historical EMS events within each administrative unit (4, 18, 19, 21), neglecting variations in EMS risk across spatial and age-based dimensions. For example, older adults individuals and those with chronic diseases might be more likely to require EMS compared to the general population, yet their distribution across geographic areas is often uneven (7). Meanwhile, even within the same population group, EMS risks may vary across different areas, influenced by factors such as environmental conditions and socioeconomic status (12). Although Li and Jia (22) proposed a model to measure spatial accessibility that considered EMS age-based risk, their approach did not consider the spatial variation in EMS risk. By failing to account for such variations in EMS demand risk, existing models may misrepresent the true spatial and demographic distribution of EMS demand, leading to inefficient EMS planning in the future.

This study aims to improve the widely used accessibility measurement and spatial optimization approaches, incorporating variations in EMS risk across both spatial and age-based dimensions. The aim can be achieved through three objectives. First, based on EMS historical data, this study develops a method to measure EMS risk across both spatial and age-based dimensions. Second, the variations in EMS demand risk are incorporated with existing accessibility and spatial optimization approaches. Third, this study compares the results between the traditional model and the improved model, identifying the differences and similarities, and discussing the importance of considering the variations in EMS demand risk.

The remainder of this paper is organized as follows. Section 2 reviews spatial accessibility measurements and related optimization models, with particular attention to applications in EMS planning. Section 3 introduces improved accessibility and optimization models. Section 4 reports the empirical study, while Section 5 provides a detailed discussion of the findings. Finally, Section 6 concludes the paper and outlines the main findings.

## 2 Literature review

### 2.1 Methodology for healthcare accessibility

Geographical studies have generally concentrated on assessing potential spatial accessibility of healthcare facilities. Over the past decades, various types of measures for evaluating healthcare accessibility have been proposed, such as proximity-based methods and the provider-to-population ratio (PPR), which focus on geographic impedance (i.e., travel distance/time) and resource availability (e.g., number of doctors, hospital beds per person), respectively (14). Gravity models integrate the above two models, considering both service availability and the impedance of distance or travel time between the population and the healthcare facilities (23).

Two-Step Floating Catchment Area (2SFCA) method, a specific case of gravity models, has been widely applied in measuring EMS accessibility (24). Many studies have improved the 2SFCA method from different dimensions, such as distance decay function [e.g., (25, 26)], competition between different service providers (15), real-time traffic conditions (27), behavior of demand (5, 28), building height (29), demand volume (30) or potential risk (22). Shin and Lee (31) added a weight of travel time into distance decay functions to estimate region-specific patient mobility. Kim et al. (32) involved the rational agent access model into the 2SFCA to better understand scene-to-hospital accessibility.

### 2.2 Methodology for spatial optimization

In healthcare planning, spatial optimization research aims to find best locations for healthcare facilities (e.g., EMS stations) or/and deploy optimal spatial distribution for healthcare resources (e.g., ambulances). The spatial optimization models can be classified into three different types, including coverage models, median problem and center problem (17). The Maximal Covering Location Problem (MCLP)—one type of coverage, and median problems are widely used to improve the system performance of EMS. The former aims to maximize the number of demands covered within a predefined service standard (e.g., 5 km or 12 min) by locating a limited number of facilities (33). The latter seeks to minimize the total travel distance/time between each demand and its nearest open facility (34).

Recently, many studies have developed numerous extensions of traditional spatial optimization models to fit the nature of EMS, such as considering different types of EMS facilities (4), modified service coverage (e.g., expected coverage, backup coverage) (21, 35), balancing EMS efficiency and equality (20) or incorporating various uncertainties (e.g., busyness rate, survival rates) (36). For instance, the MCLP model was extended to a quadratic programming model to balance the equality and efficiency of the EMS (19). Similarly, Luo et al. (20) provided a multi-objective model that integrated both MCLP and P-median models, to trade off EMS efficiency and equality. Han et al. (18) incorporated historical EMS records with the Monte Carlo simulation to simulate spatial distribution of EMS demand.

### 2.3 Application in healthcare planning

Recently, many studies have indicated spatial inequalities in healthcare accessibility, especially in the EMS sector. First, many studies have indicated uneven spatial inequalities in healthcare accessibility across different regions (3, 37). Healthcare accessibility disparities exist across both physical and virtual spatial contexts (38). Meanwhile, scholars also indicated healthcare accessibility is different from various demographic groups (39–41). For instance, McCrum et al. (40) indicated that minority groups in the United States, were most likely to experience low EMS access.

A common feature of accessibility analysis and spatial optimization models lies in their reliance on accurately characterizing the spatial distributions of healthcare demand. Recently, the majority of studies have relied on census or GPS-based data, approximating potential EMS demand by using the total population of each administrative unit [e.g., (4, 5, 29)], and thus tend to allocate EMS facilities in densely populated areas. With the advancement of medical statistical systems, some studies have begun to incorporate real historical EMS records to depict regional demand [e.g., (18)]. However, these efforts largely remain at the aggregate level and fail to capture the heterogeneity of demand among different population groups, particularly between vulnerable populations and others. Although Li and Jia (22) integrated age-specific EMS risks into the 2SFCA model, they did not account for the spatial heterogeneity of EMS risks. Against the backdrop of data-driven approaches and precision-oriented policies, urban health planning must simultaneously address the EMS risks across different regions and population groups. Therefore, it is necessary to integrate spatial variations of age-based EMS risk into existing accessibility measures and spatial optimization models, thereby generating more accurate evidence to support decision-making.

## 3 Methodology

### 3.1 Measure spatial and demographic EMS risk

Given that EMS risk may exhibit substantial variations across space and age groups, EMS risk ( $R_{i}^{a}$ ) for age group *a* at demand location *i* is evaluated based on the following notation and Equations 1, 2:

- *k*, *K*: Index and set of historical EMS records;
- *a*, *A*: Index and set of age groups;
- *c*, *C*: Index and set of emergency categories;
-
$d_{\mathrm{ik}}$ : The travel distance/time between demand location *i* and EMS record *k*;
-
$w_{c}$ : Weight of each emergency category;
- *S*: Service coverage threshold (e.g., 12-min travel time);
- *α*: Set of demand locations having at least one EMS event within coverage standard *S*;
-
$P_{i}^{\mathrm{ac}}$ : Total population of age *a* and emergency category *c* within the service coverage threshold of demand location *i*;
-
$E_{i}^{\mathrm{ac}}$ : Total historical EMS records for age *a* and emergency category *c* group within the service coverage threshold of demand location *i*;
-
$R_{\min}^{a}$ : The minimum EMS risk for age *a* group;
-
$R_{i}^{a}$ : The EMS risk for age *a* group at demand location *i*.

$$R_{i}^{a}=\frac{\sum_{c}w_{c}E_{i}^{\mathrm{ac}}}{P_{i}^{\mathrm{ac}}}\forall i\in \alpha$$ (1)

$$R_{i}^{a}=R_{\min}^{a}\;\forall i\notin \alpha$$ (2)

The evaluation of EMS risk is divided into two situations, as represented by Equations 1, 2. First, when at least one historical EMS record is located within the coverage threshold *S* of demand location i(iα) , the EMS risk for age group *a*, denoted as $R_{i}^{a}$ . When no historical EMS record is located within the coverage area centered on demand location i(iα) , the EMS risk for age group *a* is assigned to the minimum observed EMS risk for that age group, denoted as $R_{\min}$ .

### 3.2 EMS risk-based accessibility measures

This section aims to modify the most popular method for measuring healthcare accessibility—2SFCA model (24), incorporated with the variation in EMS risk influenced by spatial and demographic dimensions. The traditional model of 2SFCA model is shown in Equation 3 with the following notation.

- *i*, *e*: Index of demand location;
- *j*: Index of EMS station;
- *n*: The total number of EMS stations;
-
$S_{j}$ : Supply capacity (e.g., the number of ambulances) at the *j* th EMS station;

$$f(d_{\mathrm{ij}})\{\begin{array}{c} 1\;\;\text{if}\;d_{\mathrm{ij}}\leq S\\ 0\;\;\;\text{otherwise} \end{array}$$

$$A_{i}=\sum_{j\in (d_{\mathrm{ij}}\leq S)}[\frac{S_{j}f(d_{\mathrm{ij}})}{\;\;\;\sum_{e\in (d_{\mathrm{ej}}\leq S)}P_{e}f(d_{\mathrm{ej}})}]$$ (3)

where the first step is based on facility site. Each EMS station is assigned to a service coverage (*S*). The supply capacity of EMS station *j* (i.e., the number of ambulances) is divided by the population inside that area to get the PPR. The second step is based on demand site, the PPRs of EMS stations within the coverage are added up to compute the accessibility score at demand location *i*.

The nature of 2SFCA is the ratio-based method for measuring accessibility that accounts for both service capacity and demand competition. However, existing models have not fully accounted for the spatial and demographic variations in EMS risk, which may lead to inaccurate demand estimation. To address this issue, this study incorporates the varying characteristics of EMS risk into the 2SFCA model, based on Equation 4.


$$A_{i}=\sum_{j\in (d_{\mathrm{ij}}\leq S)}[\frac{S_{j}f(d_{\mathrm{ij}})}{\;\;\;\sum_{e\in (d_{\mathrm{ej}}\leq S)}\sum_{a\in A}R_{e}^{a}P_{e}^{a}f(d_{\mathrm{ej}})}]\;\;$$ (4)

In the modified 2SFCA model, the potential demand for *a* age-group is evaluated with EMS risk ( $R_{e}^{a})$ and population ( $P_{e}^{a})$ . The EMS risk can vary through different age groups and demand locations incorporated with Equations 1, 2.

### 3.3 EMS risk-based spatial optimization measures

The MCLP model and P-median problem are widely used spatial optimization approaches to improve EMS efficiency. Based on optimizing the limited number of EMS facilities, the former aims to maximize the number of potential demands within the coverage (*S*), and the latter seeks to minimize the weighted total travel distance/time between each demand location and its nearest EMS station.

With the following notation:

- *I, J*: Sets of locations for demand and EMS stations, respectively;
- *M*: the number of existing stations that remain open;
- *N*: the total number of stations that should be opened;
- *β*: Set of existing stations;
-
$X_{j}=\{\begin{array}{cc} 1 & \text{if}\;\mathrm{EMS}\;\text{station}\;j\;\text{is opened}\\ 0 & \text{otherwise} \end{array}$ ;
-
$Y_{\mathrm{ij}}$ : Whether the station *j* is allocated to the demand location *I*;

The formulations of two models are shown below:

Objectives:


$$\text{MCLP}:\mathrm{Maximize}:Z_{1}^{\text{mclp}}=\sum_{j\in J}\sum_{i\in I}P_{i}f(d_{\mathrm{ij}})Y_{\mathrm{ij}}$$ (5)

$$P-\text{median}:\mathrm{Minimize}:Z_{1}^{\text{median}}=\sum_{j\in J}\sum_{i\in I}P_{i}d_{\mathrm{ij}}Y_{\mathrm{ij}}$$ (6)

Subject to:


$$\sum_{j}Y_{\mathrm{ij}}=1\;\forall i\in I$$ (7)

$$\sum_{j\in \beta }X_{j}=M$$ (8)

$$\sum_{j}X_{j}=N$$ (9)

$$Y_{\mathrm{ij}}\leq X_{j}\;\forall i\in I,j\in J$$ (10)

$$X_{j}\in (0,1)\;\;\;j\in J$$ (11)

$$Y_{\mathrm{ij}}\geq 0\;\forall i\in I,j\in J$$ (12)

Equations 5, 6 represent objective for the MCLP and P-median problem, respectively. Constraints (7) ensure that each demand location should be allocated to a station *j*. Constraint (8) defines the *M* existing stations should be remained open in the system, and constraint (9) ensures that the total number of stations in the system is equal to *N*. Constraints (10) guarantee that the station *j* cannot be allocated to the demand only if the station is open (i.e., $X_{j}=1$ ). Constraints (11, 12) define the decision variables.

To incorporate the variation in EMS risk in space and age groups into the spatial optimization approaches, this study modifies the objectives as below.

Improved MCLP:


$$\mathrm{Maximize}:Z_{2}^{\text{mclp}}=\sum_{j\in J}\sum_{i\in I}\sum_{a\in A}R_{i}^{a}P_{i}^{a}f(d_{\mathrm{ij}})Y_{\mathrm{ij}}$$ (13)

Improved P-median:


$$\mathrm{Minimize}:Z_{2}^{\text{median}}=\sum_{j\in J}\sum_{i\in I}\;Z_{2}=\sum_{a\in A}R_{i}^{a}P_{i}^{a}d_{\mathrm{ij}}Y_{\mathrm{ij}}$$ (14)

where the improved MCLP involves objective (13) with constraints (1, 2, 7–12). The improved P-median problem involves the objective (14) and the constraints (1, 2, 7–12).

## 4 Empirical study

### 4.1 Research framework

**Figure 1:**
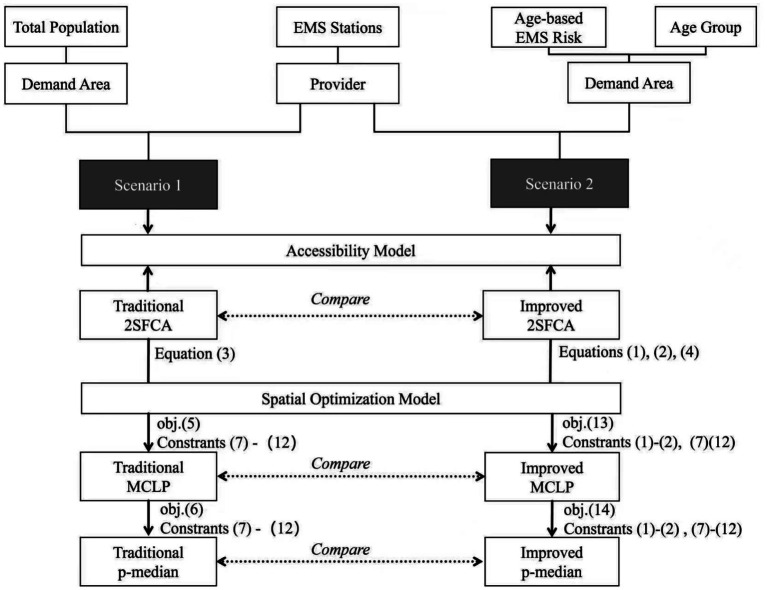
Research framework of this study.

The research framework of this study is illustrated in Figure 1, which integrates accessibility measurement with spatial optimization models under two scenarios. In Scenario 1, accessibility is measured using the traditional 2SFCA method (Equation 3), while in Scenario 2, an improved 2SFCA model incorporating factors of EMS risk variations in spatial and demographic dimensions (Equations 1, 2, 4) is applied. Then, two classic spatial optimization models—MCLP (Equation 5, Constraints 7–12) and P-median (Equation 6, Constraints 7–12)—are employed. Both models are further extended into improved versions that incorporate EMS risk (Equations 13, 14, Constraints 1, 2, 7–12). Finally, comparative analyses are conducted between the traditional and improved forms of 2SFCA, MCLP, and P-median models, in order to evaluate the impacts of integrating risk into accessibility measurement and optimization.

### 4.2 Study area

This research selects Wuhan, the capital of Hubei Province, China, as the case study area. Wuhan is located at the confluence of the Yangtze River and its largest tributary, the Han River, covering an area of 8,569.2 km^2^. The city includes 13 districts, with six districts in urban areas, and seven in suburban regions (42). As one of central China’s most important industrial and commercial hubs, Wuhan has a population of approximately 13.8 million (43), representing a 29.8% increase compared to 2010. This growth has established Wuhan as the most populous city in central China and the core of the middle reaches of the Yangtze River urban cluster. Recently, Wuhan, like many major Chinese cities, faces aging population pressures. In 2024, Wuhan had 1.576 million residents aged 65 and above, accounting for 16.6% of the population, up by 8.47 percentage points since 2010, underscoring the city’s intensifying aging trend (43). In addition, the proportion of the population aged under 14 has risen by 3.03% since 2010, largely attributable to the relaxation of the one-child policy.

Wuhan Municipal Health Commission (44) established a “Healthcare Services Network”, aiming to provide convenient healthcare services to different age-groups of residents. In EMS planning, Wuhan Government (45) launched a “EMS Rapid Service Zone” project, ensuring all patients can receive emergency medical care within 12-min after calling EMS. The Wuhan Municipal Health Commission (2025) noted that current EMS accessibility has not yet fully met the established targets, and continued efforts are required to enhance system development and service quality. By 2025, Wuhan had established 107 EMS stations; however, our estimates indicate that more than 31% of residents remain outside the 12-min (approximately 5 km) service coverage area, which is consistent with the above findings.

### 4.3 Data and model setting

The data adapted in this research can be categorized into three types, including census data with different age structures, EMS historical records in 2024, and spatial dataset. First, the 2024 census data is sourced from the WorldPop,^1^ which provides high-resolution (100 m × 100 m) gridded population datasets, stratified by 5-year age groups. In this study, the attribute value of each raster centroid was allocated to its corresponding *Shequ* (the smallest administrative unit in China), thereby enabling the aggregation of population data at the *Shequ* scale (3,493 *Shequs* in Wuhan). This procedure provides a more accurate alignment with the spatial units utilized in health planning in Wuhan. According to the recommendations of the World Health Organization (46), the population is classified into five age groups from children to senior adults, which is shown in Table 1.

**Table 1:** Age group and EMS event categories.

EMS categories	Children 0–10 ages (*a* = 1)	Adolescents 11–19 ages (*a* = 2)	Young adults 20–39 ages (*a* = 3)	Middle-aged adults 40–64 ages (*a* = 4)	Senior adults 65 ages and above (*a* = 5)
I	56	94	679	2,689	6,930
II	227	324	1,613	5,978	10,507
III	3,354	7,549	32,652	37,167	46,752
IV	323	829	3,800	9,447	21,683

**Figure 2:**
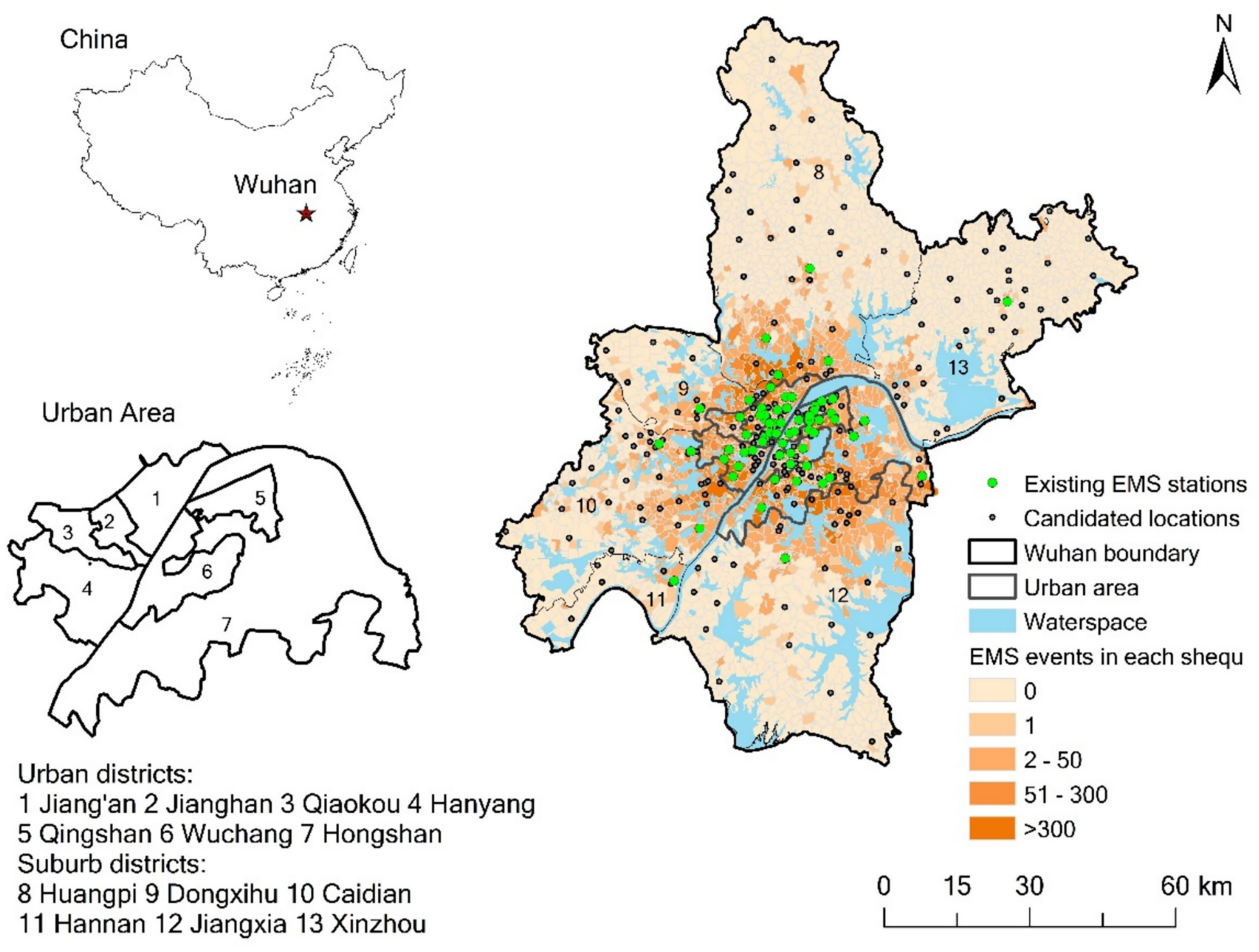
Study area and distribution of the number of EMS events in each *Shequ* in 2024.

Second, EMS historical records in 2024 were collected from Wuhan Emergency Medical Center, including 193,334 valid EMS records in Wuhan. The EMS records consist of spatial information like the patient’s location, and non-spatial attributes such as patients age or gender. Both EMS records and population data were aggregated to the *shequ* unit (Figure 2). This ensures that the numerator (EMS events) and denominator (population) refer to the same geographic area. Spatially, more than 50% of *Shequs* recorded at least one valid EMS record, primarily concentrated in the urban core and suburban districts near to the urban area. According to the classification system used by the Wuhan Emergency Medical Center, EMS records are categorized into four emergency severity levels, ranging from Level I to Level IV (*c* ∈ [1,2,3,4]). Level I refers to mild cases, while Level IV indicates the most severe and life-threatening emergencies (Table 1). Weights for different emergency categories $\left(w_{c}\right)$ were determined using an expert-scoring approach involving 10 physicians. Specifically, the weights assigned to emergency Levels I, II, III, and IV were 0.23, 0.52, 0.76, and 1.03, respectively. To better represent the spatial concentration of EMS demand within each *shequ*, this study calculated the incident-weighted centroid based on EMS call locations and used it as the representative EMS demand point for each *shequ*.

Third, spatial data includes locations of EMS stations, candidate sites for new planning stations and road network in Wuhan. In 2025, 107 EMS stations will have been operated in Wuhan, where 75 of them are distributed in the urban districts. Then, 375 hospitals are currently without EMS stations. Within this study, these facilities are designated as candidate locations for the establishment of new EMS stations, given their wide distribution across diverse areas of the city. The locations of existing EMS stations and candidate sites are collected from Amap,^2^ which is one of the largest online map services in China. The Wuhan Road Network Database of Wuhan is provided by the Wuhan Geomatics Institute,^3^ which includes road network, road level and speed limits. To construct a more operationally realistic travel-time surface, we calibrated road-network travel speeds using corrected ambulance trajectory data from approximately 13,000 EMS trips. The calibrated speeds were then assigned to the road network and used to calculate travel time between EMS stations and demand points. A 12-min travel-time threshold was adopted as service coverage.

For the model settings, the service coverage (*S*) for accessibility measurements and spatial optimization models is defined as 12-min travel time. The number of current EMS stations (*β*) is 107, and all existing EMS stations remain open in the spatial optimization models (that is M=107). Wuhan plans to establish six new EMS stations within the next 6 months; therefore, the total number of opened EMS stations in the optimization system is set to *N* = 113. For *Shequs i* that did not have any EMS events located in 2024 (*i* ∉ *α*), the EMS risk is defined as the minimum EMS risk observed among *Shequs* within EMS events.

### 4.4 Results

#### 4.4.1 Spatial distribution of age-based risk

**Figure 3:**
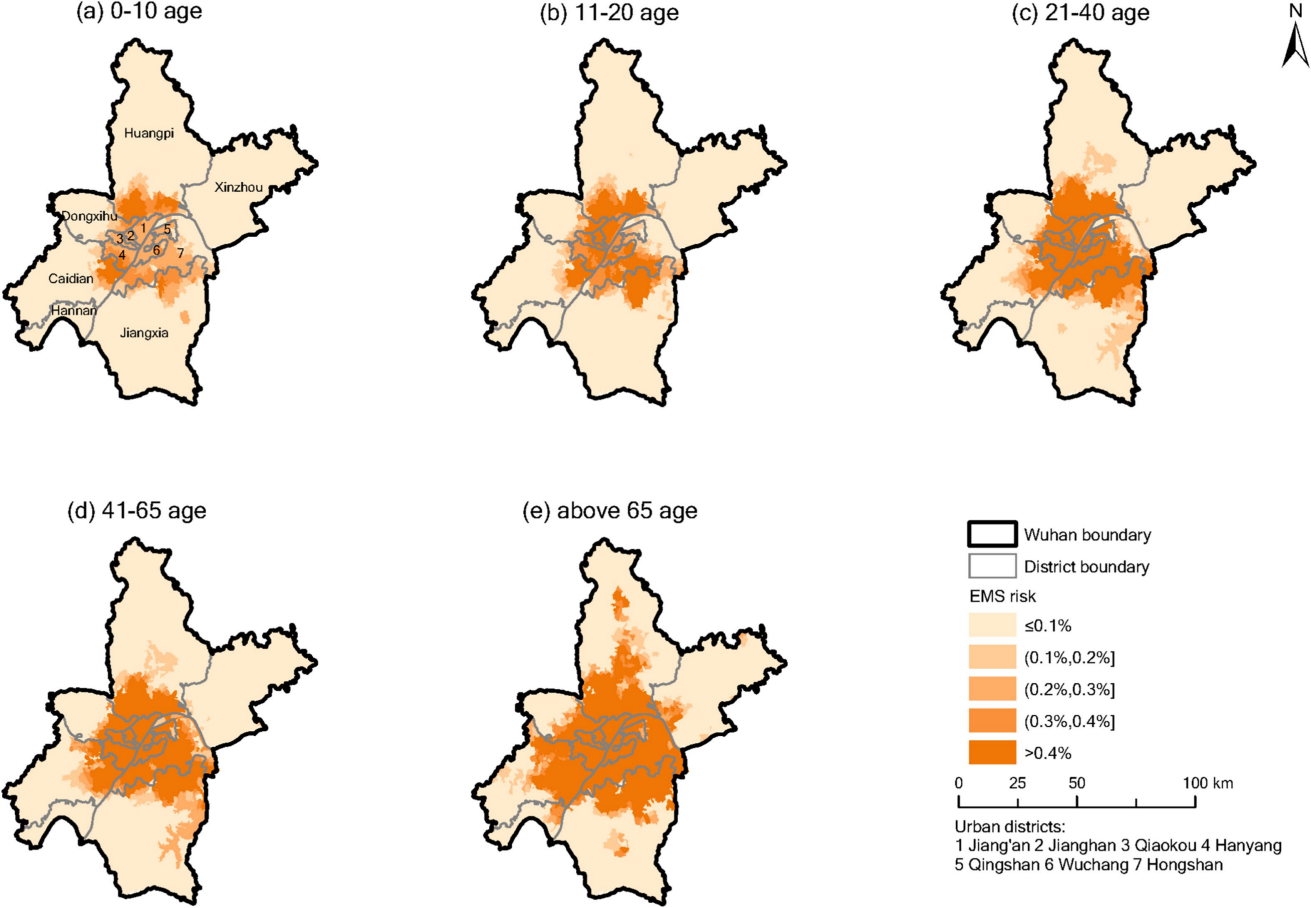
Spatial distributions of EMS risks for different age groups.

EMS risks across age groups are calculated based on Equations 1, 2, which are presented in Table 2 and Figure 3. There are marked differences in the mean EMS risks across age groups (see Table 2). In detail, the lowest mean EMS risks are observed among the 0–14 and 15–19 age groups, at 0.47 and 0.79%, respectively. Meanwhile, the highest-risk *Shequs* for the two groups, with EMS risks of 5.9 and 4.2%, are concentrated in the southwest area of Huangpi. Then, a modest increase is evident among individuals aged 20–39 and 40–64, with risks rising to 1 and 1.3%, respectively. The *Shequs* with the highest EMS risks for the above two groups is also located in the southwest area of Huangpi. By comparison, the EMS risk escalates substantially among those aged 65 and above, reaching an average of 2.5%, and the *Shequ* with the highest EMS risk (12%) for this group is also located in the southwest of Huangpi.

**Table 2:** Statistics of EMS risks among different age groups.

Age group	Mean risk	Standard deviation risk	Maximum risk
0–10	0.47%	0.6%	5.9%
11–19	0.79%	0.1%	4.2%
20–39	1%	1.5%	12%
40–64	1.3%	1.6%	11.6%
65 and above	2.5%	1.9%	12%

The spatial distribution of EMS risks for different age groups is shown in Figure 3. The distribution of EMS risk exhibits marked heterogeneity across age groups, with risks in areas near the central districts significantly higher than those in peripheral districts. Among them, the proportions of *shequs* where EMS risk exceeds 0.4% are the largest for the groups of middle-aged (40–64) and senior adults (65+), accounting for 46 and 49% of all *Shequs*, respectively. Within the urban districts, relatively high EMS risks are concentrated in central Hongshan as well as the central parts of Jiangan and Jianghan. In the peripheral districts, southern Huangpi, eastern Caidian, and northern Jiangxia show comparatively higher EMS risks. It is worth noting that, across all population groups, the EMS risk in southwestern Huangpi is markedly higher than in other areas.

#### 4.4.2 EMS accessibility

**Figure 4:**
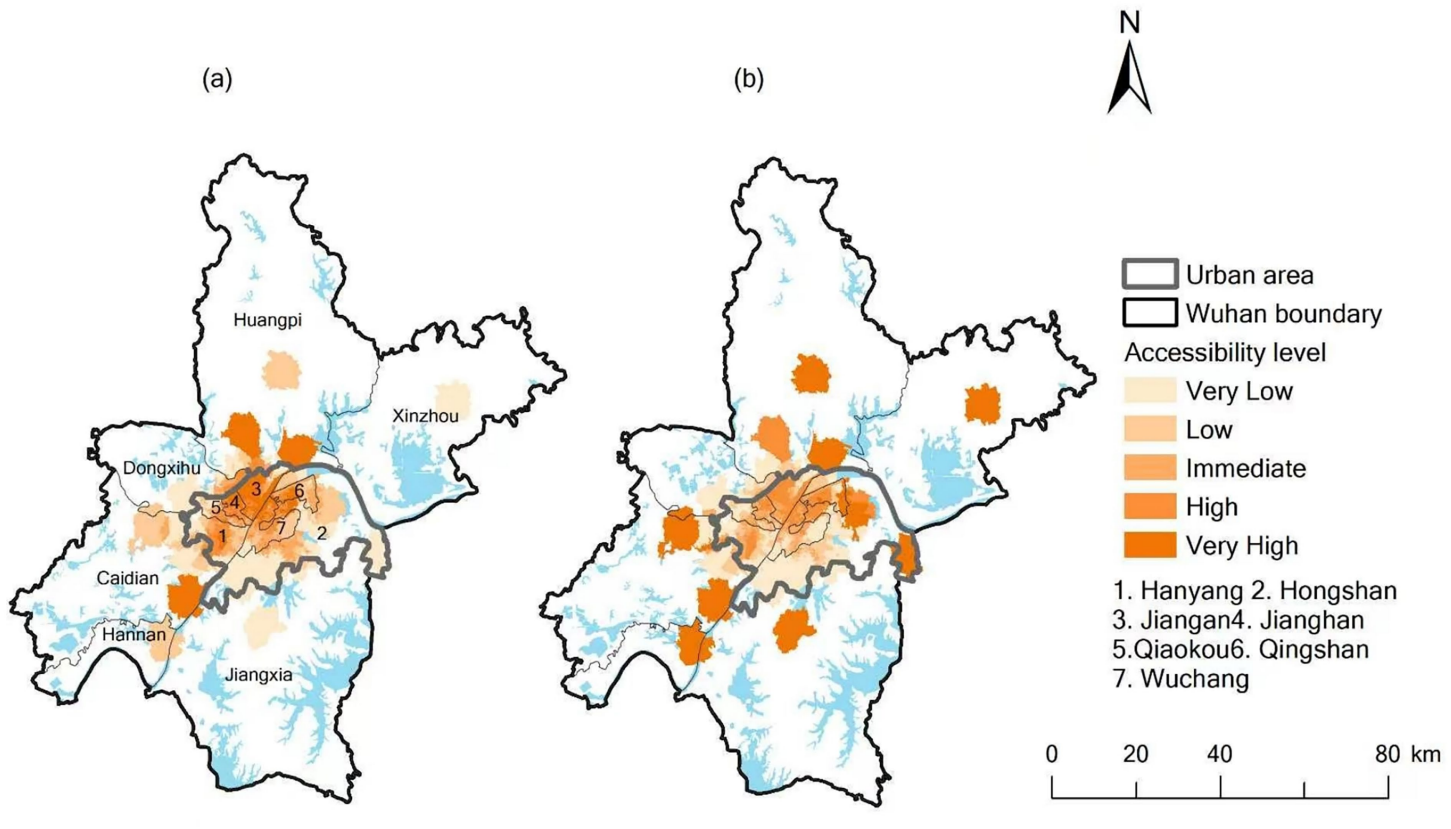
Spatial distribution of EMS accessibility: **(a)** Scenario 1; **(b)** Scenario 2.

Figure 4 presents the spatial distribution characteristics of EMS accessibility calculated based on the 2SFCA model, including two scenarios. In Scenario 1 is based on the traditional 2SFCA model without considering the age-based risk (as shown in Figure 4a). In Scenario 2, EMS accessibility is calculated based on the age-based risk 2SFCA model proposed in this study and incorporates the EMS risk of different age groups (as shown in Figure 4b). This study standardized the accessibility scores and divided accessibility into five levels using the equal-proportion grouping method, where the highest 20% of scores in each scenario are grouped into “Very high” level, and the lowest 20% values are classified into “Very low” level. Among the 3,493 *Shequs* in Wuhan shown in Figure 4, 1,522 of them are within the 12-min service area, accounting for approximately 43.5% of the city’s total *Shequs*, and about 96% of these covered *Shequs* are concentrated in core urban areas.

For these covered *Shequs*, there are significant differences in the quantity scale and spatial distribution characteristics under the two scenarios. In Scenario 1,610 *Shequs* have accessibility scores at the “Very high” level, covering 24.5% of the total population. These *Shequs* with the “Very high” level of accessibility are mainly distributed in Huangpi, Dongxihu, and Caidian Districts around the urban area (Figure 4a). In Scenario 2, 1,589 *Shequs* achieve the “Very high” level of accessibility, accounting for 23.4% of the total population. These *Shequs* are mainly distributed in Hannan and Xinzhou, a distribution quite different from that of Scenario 1.

**Figure 5:**
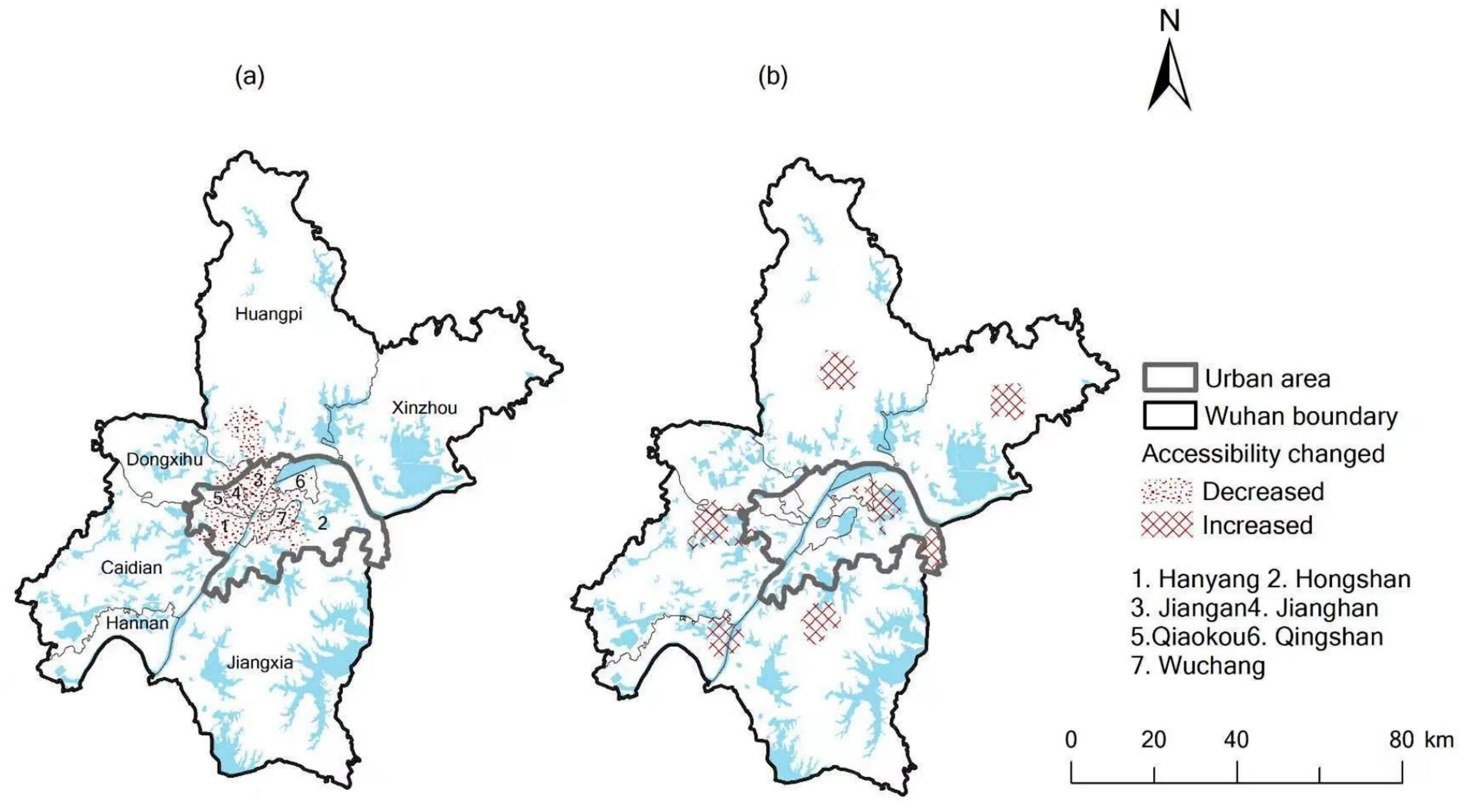
Location of Shequs with different level of EMS accessibility: **(a)** accessibility level decreased (compared to Scenario 1); **(b)** accessibility level increased (compared to Scenario 1).

The variation in EMS accessibility level between *Shequs* under two scenarios is remarkable (see Figure 5). In detail, compared to results in Scenario 1, EMS accessibility in 281 *Shequs* in Scenario 2 maintained an unchanged level, mainly distributed in urban areas, Caidian and Huangpi. Then, a total of 881 *Shequs* experience rating decreases, concentrated in urban areas and extending to southern Huangpi (Figure 5a). Meanwhile, 323 *Shequs* have rating increases, scattered across various suburban areas and Hongshan (Figure 5b).

The descriptive statistics of EMS accessibility under the two scenarios, namely the traditional 2SFCA (Scenario 1) and the improved 2SFCA (Scenario 2) are shown in Table 3. For Scenario 1, the mean accessibility value is 0.29 with a standard deviation of 0.17 and a variance of 0.028. In contrast, Scenario 2 shows a much lower mean accessibility of 0.06, accompanied by a higher standard deviation (0.18) and variance (0.32). These results suggest that, on average, the improved 2SFCA model produces smaller accessibility values but with greater variability across *Shequs*. Table 4 reports the results of Levene’s test and the independent samples t-test comparing the two scenarios. Levene’s test yields an *F* value of 190.35 (*p* < 0.001), indicating that the assumption of equal variances does not hold. Consequently, the Welch-adjusted t-test was applied. The t-test statistic is 36.49 (*p* < 0.001), demonstrating that the mean accessibility values between Scenario 1 and Scenario 2 differ significantly. This confirms that the improved 2SFCA method produces results that are statistically distinct from the traditional approach.

**Table 3:** Descriptive statistics for EMS accessibility between traditional 2SFCA (Scenario 1) and the improved 2SFCA (Scenario 2).

Scenario	*N*	Mean	Std. deviation	Variance
Scenario 1	3,493	0.29	0.17	0.028
Scenario 2	3,493	0.06	0.18	0.32

**Table 4:** Results of Levene’s test for equality of variances and independent samples t-test for Scenario 1 and Scenario 2.

Independent samples test	Levene’s test for equality of variances	t-test for equality of means		
	*F*	*t*	df	*N*
Equal variances assumed	190.35***	36.49***	2,940	1471
Equal variances not assumed		36.49***	Welch. adj	1471

***Coefficients are significant at the 0.001 level.

#### 4.4.3 Spatial optimization of new EMS stations

**Figure 6:**
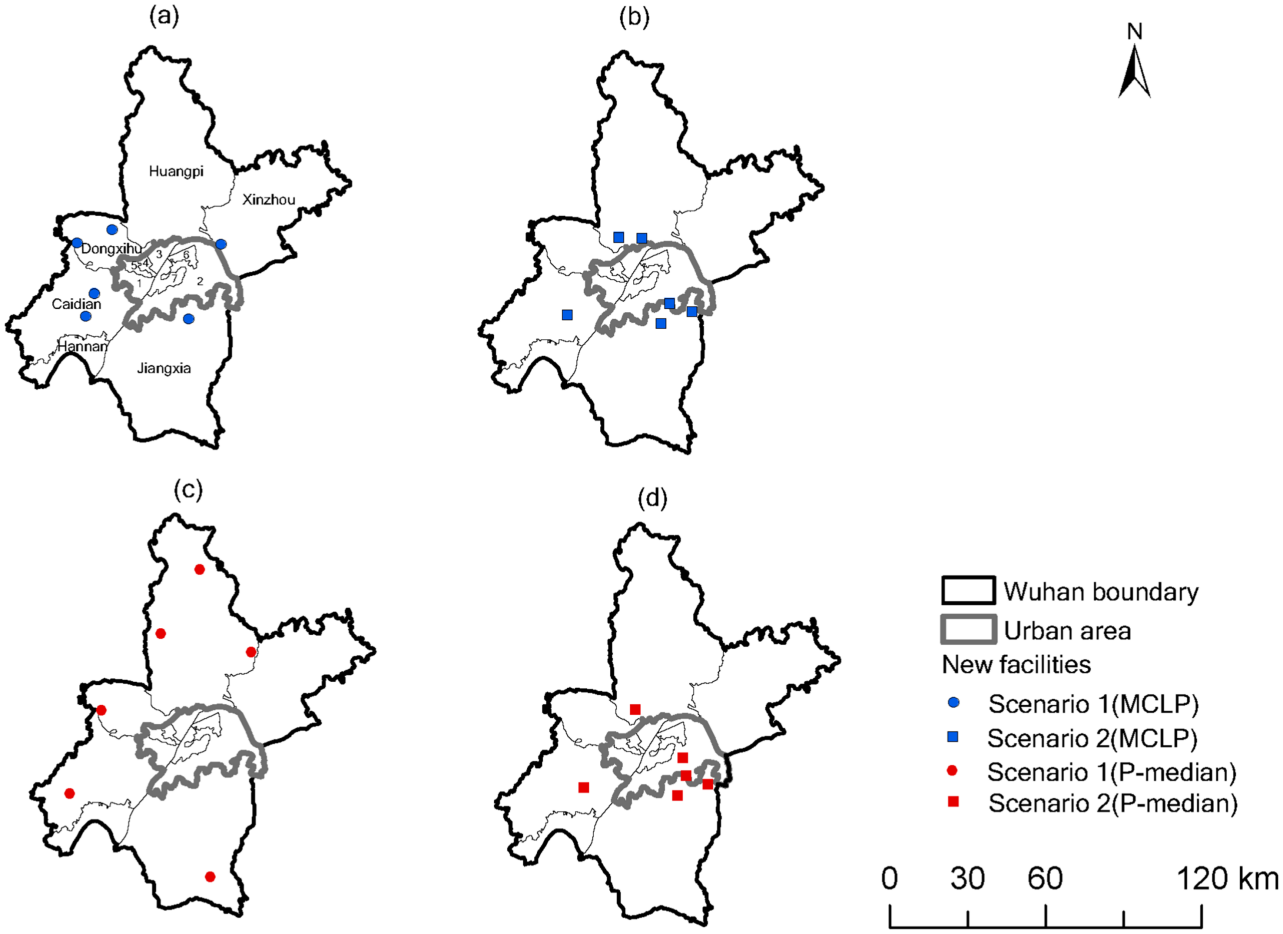
Spatial distribution of new located EMS stations in Wuhan: **(a)** traditional MCLP-based model (Scenario 1); **(b)** improved MCLP-based model (Scenario 2); **(c)** traditional P-median-based model (Scenario 1); **(d)** improved P-median-based model (Scenario 2).

Figure 6 shows the spatial distribution of six new planned EMS stations between different scenarios based on two different types of spatial optimization models. In Scenario 1, the traditional spatial optimization models would be applied, without considering age-based EMS risks. In comparison, an age-based risk model would be implemented in Scenario 2. For the MCLP model, the selected locations of new EMS stations have distinct differences between the two scenarios. In Scenario 1, the six facilities are distributed as follows: two in Dongxihu District, two in central Caidian, one in northern Jiangxia, and one in southwestern Xinzhou (see Figure 6a). In Scenario 2, two facilities are concentrated in central Huangpi, two are located in northern and northeastern Jiangxia, one in central Caidian, and one at the boundary between Jiangxia and Hongshan (see Figure 6b). In general, the two scenarios exhibit distinct logic. Scenario 1, the traditional MCLP, tends to allocate new EMS stations in the outer suburbs. However, in Scenario 2, the improved MCLP model favors locating stations in the near suburbs closer to the urban core.

Similarly, Figures 6c,d shows the optimized stations based on the P-median problem with different Scenarios. In Scenario 1, Huangpi contains three selected sites, representing the largest number across all districts. The remaining three EMS stations are situated in the western part of Dongxihu, the southwestern area of Caidian, and the southern portion of Jiangxia, respectively (see Figure 6c). In other words, all selected locations are distributed across suburban districts, dispersed in pattern, and positioned along administrative boundaries. In Scenario 2, two selected locations are distributed in the northern area of Jiangxia and the central part of Hongshan, respectively. The rest of two selected locations are distributed in the central Caidian and the southern Huangpi districts. All selected locations in Scenario 2 are near to urban districts (see Figure 6d). It can be observed that two districts (i.e., Xinzhou and Hannan) have no selected locations in either scenario, and all 6 sites differ completely between the two scenarios.

**Figure 7:**
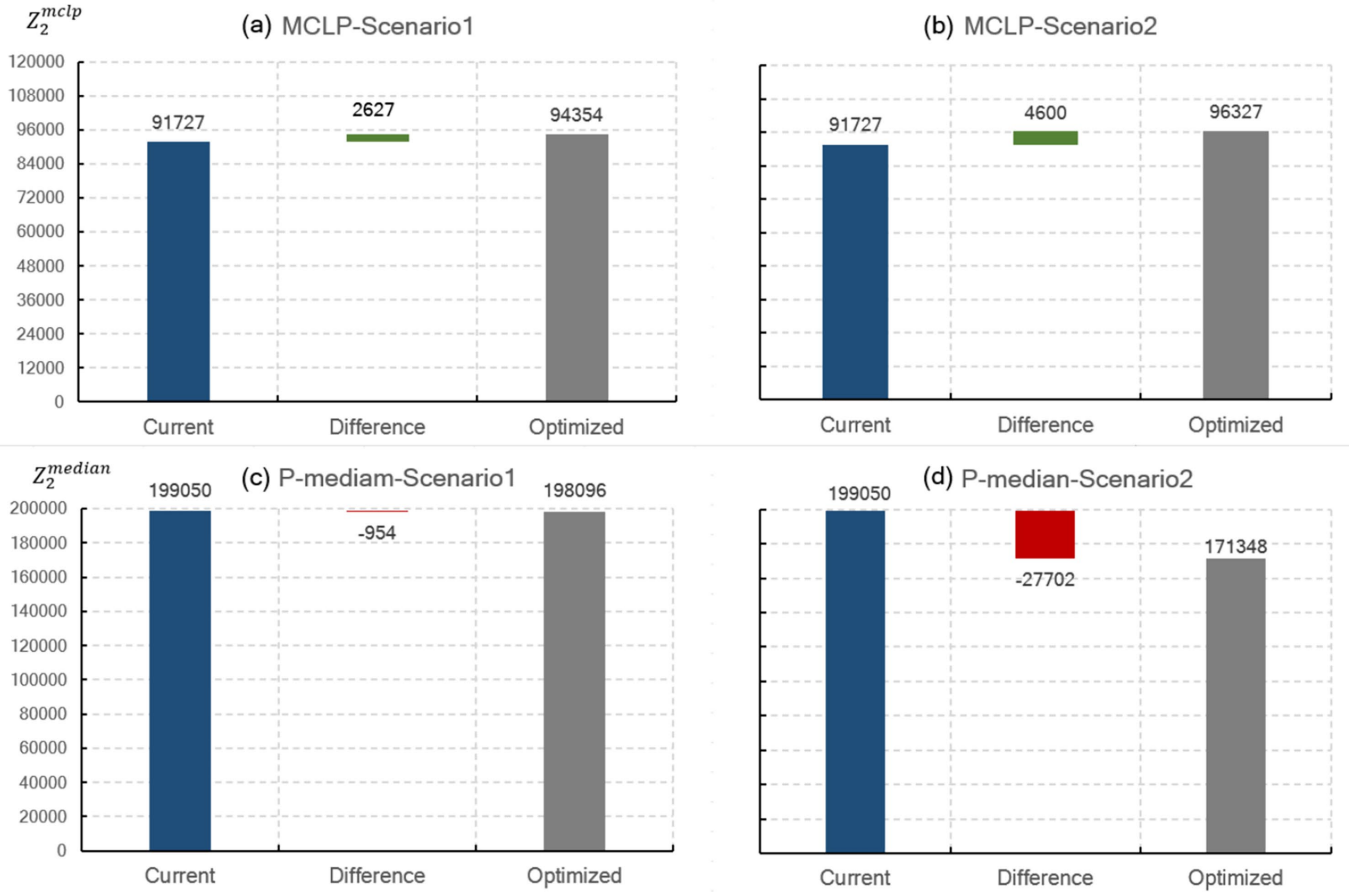
Statistical results of the traditional and improved spatial optimization models in empirical study; **(a)** Statistical results of a traditional MCLP model; **(b)** Improved MCLP model; **(c)** Traditional P-median model; **(d)** Improved P-median model in empirical study.

Figure 7 shows the difference of $\;Z_{2}^{\text{mclp}}$ and $\;Z_{2}^{\text{median}}$ under two scenarios. Using the traditional MCLP model (Scenario 1), the $\;Z_{2}^{\text{mclp}}$ value reached 91,727, representing an increase of 2,627 compared with the current situation (Figure 7a). In contrast, applying the improved MCLP model (Scenario 2) raised the $\;Z_{2}^{\text{mclp}}$ value to 96,327, an increase of 4,600 over the existing level (Figure 7b). For the P-median problem, the spatial optimization resulted in Scenario 1 was only a slight decrease of 954 compared with the current situation, representing a slight improvement in EMS accessibility (Figure 7c). In comparison, the optimized spatial layout in Scenario 2 led to a dramatic reduction in $Z_{2}^{\text{median}}$ value (27,702) compared to the current layout, showing a remarkable increase in EMS accessibility (Figure 7d). In other words, incorporating age-based risk factor into spatial optimization model leads to considerable differences in both the optimization outcomes and the corresponding strategies.

#### 4.4.4 Sensitivity analysis

To examine the influence of emergency-level weights on EMS risk estimation and research outcomes, this study conducted a sensitivity analysis. Specifically, different weight combination [*w*_1_, *w*_2_, *w*_3_, *w*_4_] were assigned to various emergency categories, and the resulting changes in EMS accessibility and spatial optimization outcomes were compared to evaluating the robustness of the model results to weight specification.

According to Table 5, the mean accessibility score remains stable across all scenarios, ranging only from 0.06 to 0.07. Specifically, when equal weights [1, 1, 1, 1], moderately adjusted weights [0.25, 0.5, 0.75, 1], and the current model weights are applied, the mean accessibility score is consistently 0.06. When more differentiated weight schemes are used, such as [0.125, 0.25, 0.5, 1] and [0.03, 0.1, 0.3, 1], the score slightly increases to 0.07. This suggests that changing the relative importance of the four criteria has only a limited influence on the overall accessibility outcome.

**Table 5:** Sensitivity analysis of emergency-level weights on EMS accessibility and spatial optimization outcomes.

Value of $\left[w_{1};w_{2};w_{3};w_{4}\right]$	Mean accessibility score	Selected EMS ID MCLP	Selected EMS ID P-median
[1, 1, 1, 1]	0.06	121, 136, 150, 238, 285, 301	136, 150, 154, 238, 285, 301
[0.25, 0.5, 0.75, 1]	0.06	121, 136, 150, 238, 285, 301	136, 150, 154, 238, 285, 301
[Current Model]	0.06	136, 142, 150, 238, 285, 301	136, 142, 150, 238, 285, 301
[0.125, 0.25, 0.5, 1]	0.07	121, 136, 150, 238, 279, 301	136, 142, 150, 238, 255, 301
[0.03, 0.1, 0.3, 1]	0.07	121, 136, 150, 238, 279, 301	136, 142, 150, 238, 259, 301

In terms of spatial optimization outcomes, the MCLP model shows a highly consistent selection pattern. EMS stations 121, 136, 150, 238, and 301 are repeatedly selected across all scenarios, indicating that these stations are structurally important for maximizing coverage. Only one selected station changes under lower-weight scenarios, with station 285 being replaced by 279. The P-median model also shows a relatively stable pattern, with EMS stations 136, 150, 238, and 301 consistently selected in all scenarios. However, compared with MCLP, the P-median results are slightly more sensitive to weight changes. For example, station 154 is selected under the first two scenarios, while stations 142, 255, and 259 appear under alternative weight combinations.

## 5 Discussion

This study improves the existing accessibility and spatial optimization models through the integration of spatial and age-related variations in EMS risks. The empirical analysis reveals distinct age-related and spatial variations in EMS risk in Wuhan. EMS risk increases steadily with advancing age, with individuals aged 65 and above facing remarkably higher EMS risks than other groups. Regarding spatial heterogeneity, EMS risk is generally higher in central urban areas than in suburban districts, and the spatial disparity becomes more pronounced with increasing age.

Compared to the results of traditional models, the improved models show different EMS accessibility patterns and optimized layouts. In accessibility analysis, using the traditional 2SFCA model without accounting for age-based risks in Scenario 1, *Shequs* with “very high” level of EMS accessibility were mainly concentrated in the north of urban districts, such as Jiangan or Qiaokou. Once integrated the EMS age-based risks in Scenario 2, areas with “high accessibility” level shifted toward the outer suburbs where close to EMS stations. In spatial optimization, when age-based risks were excluded in Scenario 1, both the traditional MCLP and P-median models tended to recommend new EMS stations in outer suburbs. In contrast, when EMS risk-based spatial optimization models were applied in Scenario 2, the results favored placing the six planned EMS stations in near-suburban areas closer to the urban boundary.

The research found that EMS risk increases significantly with age, which is consistent with many previous studies (11). EMS risk exhibits pronounced spatial variation, may be jointly influenced by multiple factors. Due to the marked differences in population density and age structure across urban areas, spatial variations in EMS risk are evident in many places. In addition, variations in the built environment, such as road networks, land-use functions, and building characteristics, also influence both the frequency of EMS demand and the efficiency of EMS response. It should be noted that the EMS risk estimated in this study represents realized EMS demand risk derived from historical records, rather than a purely exogenous measure of underlying health needs. Historical EMS use may be affected by both actual emergency demand and the existing accessibility of EMS services. Therefore, the estimated risk is interpreted as an empirical measure of observed EMS utilization adjusted by age-group population and emergency severity.

The choice of incident volume and EMS risk rather than simple population coverage is grounded in the specific nature of EMS planning. Population coverage reflects the potential number of residents who may benefit from EMS access, whereas incident volume captures realized emergency demand and the spatial heterogeneity of actual EMS use. In EMS systems, service need is not proportional to population size alone, because different communities/*Shequs* and demographic groups may have different probabilities of requiring emergency care due to age structure, health status, built environment, socioeconomic conditions, and institutional concentrations of vulnerable populations. Therefore, an incident-based objective is methodologically suitable for identifying locations where EMS resources are most likely to reduce actual service burden and response delays. Philosophically, this approach follows a needs-based planning logic: limited EMS resources should not only be allocated to where more people live, but also to where emergency needs are empirically concentrated. However, this does not imply that population coverage is unimportant or that incident volume is universally superior. Rather, population-based indicators remain essential for evaluating broader spatial equity, while incident volume provides a complementary measure of realized demand risk. To avoid scale-driven bias between efficiency and equity, a multi-objective optimization framework, such as simultaneously maximizing incident-based EMS coverage and minimizing the population remaining outside the service coverage threshold. Such an extension would allow the model to better balance demand responsiveness and spatial equity, thereby reducing the risk that marginalized or underserved areas are systematically overlooked.

This study yields several policy implications. First, since EMS risk is disproportionately higher among senior adults, healthcare decision-makers should explicitly incorporate age structure into EMS planning to ensure equitable and timely access for the older adults. Given the elevated EMS risk in urban districts and their surroundings, priority should be placed on allocating more EMS resources in these areas to reduce demand pressure and improve response efficiency. Second, the fact that over 35% of *Shequs* remain outside EMS service coverage reveals a critical gap in resource allocation. To address this, the government should expand investment in EMS resources and establish new stations in suburban and underserved areas, thereby enhancing both equity and accessibility. Third, future EMS planning should more effectively integrate real EMS data to better capture regional disparities and improve the precision of accessibility assessments and spatial optimization. For example, models based solely on basic population data (Scenario 1) tend to recommend new stations in peripheral areas of Wuhan, whereas incorporating EMS big data and population characteristics (Scenario 2) yields results that favor locating new stations in central urban districts and their surroundings.

Some limitations of this study should be acknowledged. First, the analysis did not incorporate disease-specific classifications or severity levels. Medical conditions vary in urgency and treatment needs, and overlooking this heterogeneity may reduce the precision of EMS demand assessment and limit the relevance of policy recommendations. Second, EMS accessibility was measured using road network distance rather than travel time. Distance provides a basic proxy for spatial separation, but it fails to capture the effects of traffic congestion, road conditions, and speed limits that directly shape emergency response efficiency. Third, the candidate sites for new EMS facilities were restricted to existing hospitals, without considering alternative locations such as vacant land, public service centers, or transportation hubs. This constraint may limit the flexibility of the optimization model and reduce the practical applicability of the results. Moreover, although the proposed model incorporates spatial and age-based variations in EMS risk, it does not explicitly distinguish the independent effects of ecological environment, socioeconomic status, built environment, disease prevalence, population mobility, or temporal fluctuations. These factors may jointly shape the observed spatial variation in EMS demand and are therefore partially reflected in the historical EMS records used in this study.

Further research can be discussed. First, future research could integrate external indicators of underlying health needs, such as disease incidence, mortality records, emergency department visits, chronic disease prevalence, and socioeconomic vulnerability. Such extensions would help reduce potential circularity between accessibility and EMS use and provide a more robust basis for EMS planning. Second, future research could further incorporate second-due or backup coverage into the proposed optimization framework, especially for high-severity EMS demand. In this study, EMS records were classified into four emergency levels, with Level IV representing the most critical and life-threatening cases. For these Level IV emergencies, reliance on only primary coverage may be insufficient, as the nearest ambulance or station may be unavailable. Future models could require high-risk locations, particularly areas with frequent Level IV cases, to be covered by both primary and secondary EMS stations. In addition, future research could extend the proposed framework by explicitly incorporating additional determinants of EMS demand, such as socioeconomic indicators, land-use characteristics, environmental exposure, population mobility, and temporal patterns.

## 6 Conclusion

This study improves EMS accessibility and spatial optimization models by incorporating spatial and age-related variations in EMS risks. Evidence from Wuhan shows that EMS risk rises significantly with age, especially among older adults populations, and exhibits pronounced spatial heterogeneity shaped by demographic and built-environment-related factors. Compared with traditional approaches, the improved models generate distinct accessibility patterns and station layouts. Conventional models favored outer suburban allocations, whereas risk-based models shifted optimal sites toward near-suburban areas closer to the urban core. These differences highlight the importance of capturing demand heterogeneity for more effective EMS planning. Policy implications include prioritizing services for older adults groups and high-risk urban districts while addressing coverage gaps in underserved suburban areas. Integrating EMS big data with demographic structures can yield more precise planning and support equitable, resilient systems. Overall, this framework provides stronger evidence for EMS decision-making and offers transferable insights for rapidly urbanizing and aging regions.

## Data Availability

The data analyzed in this study is subject to the following licenses/restrictions: the datasets used and analyzed during the current study are available from the corresponding author on reasonable request. Requests to access these datasets should be directed to weicongluo@mail.hzau.edu.cn.

## Footnotes

1
https://www.worldpop.org/

2
https://lbs.amap.com/

3
https://whkc.com.cn/
